# The accuracy of screening tools for sarcopenia in older Chinese adults: a systematic review and meta-analysis

**DOI:** 10.3389/fpubh.2024.1310383

**Published:** 2024-02-05

**Authors:** Siyu Qian, Siqing Zhang, Mengchen Lu, Shuhang Chen, Liyao Liu, Siqi Liu, Fanglin Jiang, Jisheng Zhang

**Affiliations:** ^1^School of Physical Education, Hunan Normal University, Changsha, China; ^2^School of Physical Education, Huazhong University of Science and Technology, Wuhan, China

**Keywords:** sarcopenia, screening tool, older Chinese adults, accuracy, systematic review and meta-analysis

## Abstract

**Objective:**

This review aimed to analyze and compare the accuracy of eight screening tools for sarcopenia in older Chinese adults according to different diagnostic criteria.

**Methods:**

This systematic review was conducted in accordance with the Preferred Reporting Items for Systematic Reviews and Meta-Analyses guidelines. The PubMed, Embase, Web of Science, China National Knowledge Infrastructure (CNKI), and Wanfang databases were searched between the publication of the first expert consensus on sarcopenia in 2010 and April 2023 using relevant MeSH terms. We evaluated the risk bias of the included studies using the Quality Assessment of Diagnostic Accuracy Studies-2 (QUADAS-2) tool. The pooled result of sensitivity, specificity, positive likelihood ratio (PLR), negative likelihood ratio (NLR), diagnostic odds ratio (DOR), and plot the summary receiver operating characteristic curve (SROC) were calculated by using a bivariate random-effects model. The accuracies of sensitivity and specificity of the screening tools were compared using the Z-test.

**Results:**

A total of 30 studies (23,193 participants) were included, except for calf circumference (CC), Ishii, and Finger-ring Test; Screening tools for sarcopenia in older Chinese adults have consistently shown low to moderate sensitivity and moderate to high specificity. Regional and sex differences affect the accuracy of the screening tools. In terms of sensitivity and specificity, the CC, Ishii, and Finger-ring Test were superior to the other screening tools.

**Conclusion:**

The Asian Working Group on Sarcopenia (AWGS) 2019 criteria are more appropriate for the diagnosis of sarcopenia in older Chinese adults. According to the AWGS 2019, CC and Ishii are recommended for sarcopenia screening in older Chinese adults.

## 1 Introduction

Sarcopenia is a disease which seriously harms the physical health of older adults (1), resulting in reduced activity capacity, increased risk of falls, aggravated disability, and reduced ability to perform activities of daily living. Sarcopenia is also associated with cognitive decline, hospitalization, and death (2–6). Sarcopenia progression is a dynamic process that is not easily detected in the early stages and may only be recognized when it is severe enough to cause loss of physical function, falls, and autonomy (7). The early identification of risk factors for sarcopenia and exercise interventions have been shown to be highly effective in reducing the incidence of associated adverse outcomes (e.g., falls, decreased somatic function) (8, 9), and aggressive management could reduce the prevalence of sarcopenia by 10%, which is expected to save at least $1.1 billion per year (10). Moreover, the selection of convenient and accurate sarcopenia screening tools can effectively simplify the screening process, facilitate the early identification of sarcopenia by medical personnel and researchers, and reduce the risk of its occurrence, which is of great importance for improving the quality of life of older adults.

Various screening tools for sarcopenia have been developed, including the SARC-F questionnaire (11), SARC-F combined calf circumference (SARC-CalF) questionnaire (12), Ishii score (13), Mini Sarcopenia Risk Assessment (MSRA-7/MSRA-5) (14), calf circumference (CC) (15), Finger-ring Test/ Yubi-Wakka (16), and middle upper arm circumference (MUAC) (17). The European Working Group on Sarcopenia in Older People (EWGSOP), Asian Working Group on Sarcopenia (AWGS), International Working Group on Sarcopenia (IWGS), and Foundation for the National Institutes of Health (FNIH) have published guidelines for the diagnosis and treatment of sarcopenia (15, 18–22). IWGS recommends the screening of sarcopenia using the SARC-F (21). SARC-F and Ishii were included in EWGSOP 2 of case finding (19). The AWGS 2019 recommends screening for sarcopenia using SARC-F and SARC-CalF (20). Different guidelines recommend different screening tools. If the accuracy of different screening tools can be analyzed and compared under the same diagnostic criteria, it may provide new ideas for researchers to perform sarcopenia screening in different areas, thus promoting the screening of sarcopenia in older adults.

In a study by Yang et al. (23), the sensitivity of SARC-F was highest when using the diagnostic criteria of the FNIH and lowest when using the diagnostic criteria of the IWGS. The prevalence of sarcopenia depends on the diagnostic criteria used (24), and the accuracy of the screening tools varies according to these criteria. The accuracy of the screening tools using different diagnostic criteria requires further investigation.

Several tools have been widely used for sarcopenia screening; for example, the SARC-F is easy to implement and has been validated in different populations (12, 25). However, different subject characteristics of subjects may affect the accuracy of screening tools, and several factors associated with the prevalence of sarcopenia have been reported. Yu et al. found that age, sex, and disease were related to the occurrence of sarcopenia in the Chinese population, and that differences in population, race, and living environment affected the prevalence of sarcopenia (26). A meta-analysis based on the accuracy of SARC-F screening for sarcopenia sub-grouped by population and region, showed that different populations and regions resulted in differences in the accuracy of the SARC-F screening for sarcopenia (27). Whether these risk factors affect the accuracy of the screening tools requires further investigation.

China has a growing aging population (28) and large-scale diagnosis of sarcopenia is challenging. Hence, and it is important to use a convenient tool to screen older adults for sarcopenia. China has a growing aging population and large-scale diagnosis of sarcopenia is challenging. Hence, and it is important to use a convenient tool to screen older adults for sarcopenia, our systematic review has three objectives. First, evaluating the accuracy of eight screening tools for screening sarcopenia among older Chinese adults. Second, exploring the sources of heterogeneity which may affect the accuracy of screening tools. Third, finding the screening tools and diagnostic criteria suitable for older Chinese adults, in order to provide a reference for relevant practitioners and future research.

## 2 Materials and methods

Our systematic review was conducted in accordance with the Preferred Reporting Items for Systematic Reviews and Meta-Analyses guidelines, the PRISMA checklist is available from the Supplementary material. The PICOS strategy was utilized for the inclusion criteria.

### 2.1 Literature search

The first expert consensus on sarcopenia was published in 2010 (18), until 2016 when sarcopenia was officially classified as a disease by The World Health Organization (WHO) (29), and the latest expert consensus on sarcopenia was updated in 2019 (19). Sarcopenia-related studies have been increasing and maturing after 2010, and for the literature search to be as comprehensive as possible, the PubMed, Embase, Web of Science, China National Knowledge Infrastructure (CNKI), and Wanfang databases were searched between January 2010 and April 2023. To avoid missing searches, we searched for specific screening tools in addition to the “screening tool.” The search strategy was “screening tool” or “SARC-F” or “SARC-CalF” or “Mini Sarcopenia Risk Assessment” or “MSRA” or “Finger-ring Test” or “Yubi-wakka” or “Ishii” or “CC” or “calf circumference” or “MUAC” and “sarcopenia” or “muscle mass.” Two authors independently conducted a literature search. If the two authors’ opinions differed, a third reviewer was consulted.

### 2.2 Article selection

WHO has made age boundaries for the older adults, and as China is the largest developing country in the world, the starting age standard for the older adults is 60 years old. Based on the above, the inclusion criteria developed based on the PICOS strategy are as follows: P: subjects were older Chinese adults aged ≥60 years; I: study conducted screening for sarcopenia; C: diagnostic criteria for sarcopenia were derived from EWGSOP or AWGS or FNIH or IWGS guidelines, the detailed criteria are listed in (Table 1); O: study reports the accuracy of sarcopenia screening tool, including true positive (TP), false positive (FP), false negative (FN), and true negative (TN); S: diagnostic test.

**Table 1:** Summary of operational diagnostic criteria for sarcopenia by sex.

	Diagnosis criteria			Diagnose
	1. Low muscle mass	2. Low HS (kg)	3. Low GS (m/s)	
AWGS 2014①	Male:≤7.0 kg/m2	Male:<26	<0.8	1 + 2^†^ or 1 + 3^†^
	Female:≤5.7 kg/m2	Female:<18		
AWGS 2019②	Male:<7.0 kg/m2	Male:<28	<1.0	1 + 2^†^ or 1 + 3^†^
	Female:<5.7 kg/m2	Female:<18		
EWGSOP 1③	Male:≤8.87 kg/m2	Male:<30	<0.8	1 + 2^†^ or 1 + 3^†^
	Female:≤6.42 kg/m2	Female:<20		
EWGSOP 2④	Male:<7.0 kg/m2	Male:<27	<0.8	1 + 2^†^ or 1 + 2 + 3^‡^
	Female:<5.5 kg/m2	Female:<16		
FNIH⑤	ASM/BMI Male: 0.789	Male:<26	<0.8	1 + 2 + 3^†^
	ASM/BMI Female:0.512	Female:<16		
IWGS⑥	Male:≤7.23 kg/m2	-	<1.0	1 + 3^†^
	Female:≤5.67 kg/m2			

①, Asian Working Group for Sarcopenia 2014; ②, Asian Working Group for Sarcopenia 2019; ③, European Working Group on Sarcopenia in Older People 2010; ④, European Working Group on Sarcopenia in Older People 2019; ⑤, International Working Group on Sarcopenia; ⑥, Foundation for the National Institutes of Health; HS, hand grip strength; GS, gait speed.

†, diagnosed with sarcopenia; ‡, diagnosed with severe sarcopenia.

Studies were excluded based on the following criteria: (1) meeting minutes, letters, comments, and reviews; (2) insufficient data and inability to contact the original authors; (3) subjects with major medical conditions such as diabetes, dialysis, cancer, stroke, psychiatric disorders or bone fractures; and (4) language other than English or Chinese.

Standardizing the inclusion and exclusion criteria before literature search can minimize the impact of heterogeneity on the accuracy of study results. To avoid omissions, two reviewers with systematic training independently selected the articles. If the two reviewers disagreed, a third reviewer was consulted.

### 2.3 Data extraction

The following data were extracted independently by two authors: authors, year, region, population, sample size, age, percentage of females, cutoff for screening tools, diagnostic criteria for sarcopenia, prevalence, and TP, FP, TN, and FN. If the information was insufficient, the original authors were contacted via email.

### 2.4 Quality assessment

The quality of a meta-analysis conclusions depends not only on rigorous operational procedures but also on the control of bias by the included studies. We used the Quality Assessment for Diagnostic Accuracy Studies-2 (QUADAS-2) (30) to assess the risk of bias in four dimensions: participant selection, index test, reference standards, and flow and timing, which is available.^1^ Based on responses to the relevant questions in each part, the risk level of bias could be assessed as “low,” “high” or “unclear.” Two authors independently assessed the quality of included studies. The results were presented graphically.

### 2.5 Statistical analysis

The studies were grouped according to the different sarcopenia diagnostic criteria used in the screening tool. The detailed criteria are listed in Table 1. The pooled result of sensitivity, specificity, positive likelihood ratio (PLR), negative likelihood ratio (NLR), diagnostic odds ratio (DOR), 95% confidence interval (CI), and plot the summary receiver operating characteristic curve (SROC) were calculated by using a bivariate random-effects model (31). High sensitivity indicated a low missed diagnosis rate, whereas high specificity indicated a low misdiagnosis rate (32, 33). The LR is the probability ratio of patients with and without disease, which is not affected by the prevalence rate and fully reflects the value of diagnostic tests (34). The DOR reflects the degree of correlation between diagnostic test results and diseases (35). The closer the AUC value is to 1, the better the test performance, and the higher the DOR value, the higher the AUC (36). Cochran’s Q test was used to assess the inter-study heterogeneity. The degree of heterogeneity was assessed using I^2^, with I^2^ values of 25, 50, and 75%, indicating low, moderate, and high heterogeneity, respectively (37). Z-test (38) was performed to compare the pooled sensitivity or specificity of each screening tool, in which *p* ≤ 0.05 and *p* ≤ 0.01 indicated differences and significant differences, respectively.

Bivariate random-effects models were used to correct for differences in index test thresholds (cutoff values) and between-test variations in test accuracy (heterogeneity) (39). Stata 17.0 was used for meta-analysis when there were more than four articles defining the diagnostic criteria using the guidelines, and Meta-DiSc1.4 was used for accuracy consolidation when there were fewer than four articles. Meta-regression and subgroup analyses were used to explore and explain the heterogeneity between studies based on region, population, and sex. Deeks’ funnel plot was used to evaluate publication bias, with *p* < 0.05, indicating publication bias (40).

Statistical analyses were done using Stata 17.0 (StataCorp, College Station, TX, United States) and Meta-DiSc 1.4 (Universidad Complutense, Madrid, Spain) with the “metandi” and “midas” modules.

## 3 Results

### 3.1 Description and methodological quality of the included studies

#### 3.1.1 Literature search process

**Figure 1:**
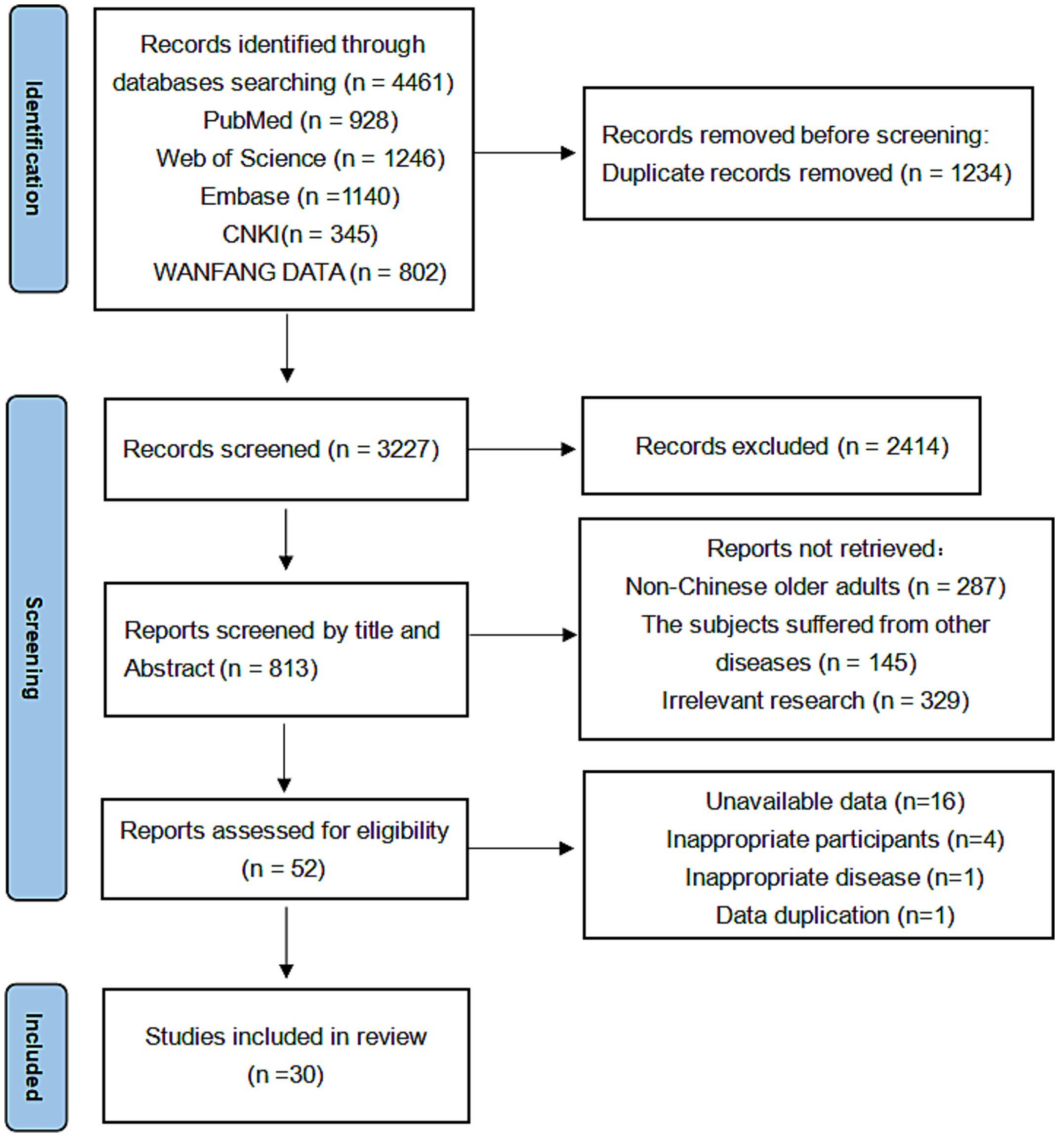
PRISMA flow chart.

A total of 4,416 records were extracted from the literature search and 1,234 duplicate records were deleted before formal screening. 52 records remained after excluding those that did not meet the criteria, of which 30 studies (23,193 subjects) met the eligibility criteria and were included in the meta-analysis (Figure 1).

#### 3.1.2 Characteristics of the included studies

We created a data extraction table based on study characteristics (Table 2). The mean age of the patients was 72.31 ± 4.06 years, and 54.15% of the subjects were female. The prevalence of sarcopenia was 11.22, 22.94, 10.63, 15.21, 21.9, and 19.62% among older Chinese adults according to the diagnostic criteria of AWGS 2014, AWGS 2019, EWGSOP 1, EWGSOP 2, IWGS, and FNIH diagnostic criteria, respectively.

**Table 2:** Characteristics of the included studies.

First Author, Year	region	Population	Sample size	Age, y	Female, %	screening tool	Cutoff	Diagnostic criteria	Prevalence, %	TP	FP	FN	TN
Yang, 2018 (23)	Cheng du	Ccommunity	384	71.5	58.33	SARC-F	4	①	15.9	18	6	43	317
								③	11.7	9	15	36	324
								⑤	25.0	19	5	77	283
								⑥	15.4	18	6	41	319
						SARC-CalF	11	①	15.9	37	17	24	306
								③	11.7	22	32	23	307
								⑤	25.0	41	13	55	275
								⑥	15.4	33	21	26	304
Lu, 2021 (41)	Yi bin	Ccommunity	588	68.8	46.77	SARC-F	4	①	10.71	49	14	14	511
Lin, 2020 (42)	Cheng du	Ccommunity	825	68.8	50.5	SARC-F	4	①	10.3	1	7	84	733
Pei Pei, 2020 (43)	Bei jing	Ccommunity	527	72.5	0	SARC-F	4	①	17.08	26	8	64	429
								④	17.46	26	8	66	427
Li, 2020 (44)	Cheng du	Ccommunity	1009	68.1	45.89	SARC-F	4	①	8.6	20	110	67	812
						SARC-CalF	11	①		36	130	51	792
Woo, 2014 (45)	Hong Kong	Ccommunity	4000	73.9	49.99	SARC-F	4	①	7.33	19	131	274	3573
							4	③	9.28	25	125	336	3511
							4	⑤	20.17	47	103	759	3088
Lin, 2021 (46)	Zi gong	Nursing home	199	NR	51.3	SARC-F	4	②	48.74	39	17	58	85
						SARC-CalF	11	②		69	40	28	62
						CC	M 34cm	②		42	17	16	22
							F 33cm	②		31	32	8	31
						Ishii	M 105	②		55	17	3	22
							F 120	②		32	9	7	54
Lin, 2023 (47)	Tai wan	Ccommunity	209	77.7	69.38	SARC-F	4	②	40.7	46	37	39	87
						SARC-CalF	11	②		65	33	20	91
						CC		②		73	39	12	85
Yang, 2018 (48)	Cheng du	Ccommunity	384	71.5	58.33	MSRA-7	30	①	15.9	53	195	8	128
						MSRA-5	45	①		55	95	6	228
Guanghui, 2020 (49)	Shang hai	Ccommunity	515	70.2	66.2	SARC-F	3	①	17.9	37	19	55	404
Li, 2019 (50)	He fei	Hospital	138	71.7	50	SARC-F	4	①	25.36	15	8	20	95
						Ishii	M 105	①		14	16	2	37
							F 120	①		15	16	4	34
Yang, 2018 (51)	Cheng du	Nursing home	277	81.6	70.03	SARC-F	4	①	34.3	19	3	76	179
								③	32.5	16	6	74	181
								⑤	38.3	18	4	88	167
								⑥	31.4	19	3	68	187
						SARC-CalF	11	①	34.3	56	26	39	156
								③	32.5	53	27	37	160
								⑤	38.3	59	23	47	148
								⑥	31.4	56	26	31	164
						MSRA-7	30	①	34.3	54	31	41	151
								③	32.5	48	37	42	150
								⑤	38.3	58	27	48	144
								⑥	31.4	50	35	37	155
						MSRA-5	45	①	34.3	51	29	44	153
								③	32.5	46	32	44	155
								⑤	38.3	52	28	54	143
								⑥	31.4	49	31	38	159
Zhou, 2022 (52)	Lu zhou	Ccommunity	439	70.51	50.4	SARC-F	4	②	26.43	13	13	93	282
						SARC-CalF	11	②		50	25	56	270
						SARC-F	4	④	12.5	10	16	40	335
						SARC-CalF	11	④		28	47	22	304
Yihan, 2021 (53)	Chang sha	Ccommunity	202	70.9	47.52	CC		②	22.8	41	34	5	122
Chen, 2022 (54)	Tai wan	Ccommunity	177	78.7	47.46	SARC-F	4	②	51.98	10	7	82	78
						SARC-CalF	11	②		35	17	57	68
						MSRA-5	45	②		56	39	36	46
						CC		②		74	24	18	61
Pengtian, 2021 (55)	Shijiazhuang	Ccommunity	303	78.35	63.04	Finger-ring Test		②	26.4	56	32	24	191
Xiaoyan, 2021 (56)	Lu liang	Ccommunity	1455	70.97	52.1	SARC-F	4	②	18.69	58	163	214	1020
						SARC-CalF	11	②		181	86	91	1097
Zhu, 2022 (57)	Zi gong	Nursing home	199	75.17	51.26	SARC-F	2	②	33.7	57	59	10	73
						SARC-CalF	12	②		46	37	11	95
						Ishii	M 130	②		34	13	6	44
							F 130	②		26	9	1	66
Pengtian, 2020 (58)	Shijiazhuang	Ccommunity	303	68	63.1	SARC-F	4	②	24.42	51	47	23	182
						Finger-ring Test		②		56	32	18	197
						MSRA-5	45	②		46	43	28	186
Youping, 2021 (59)	Yi bin	Ccommunity	503	68.4	46.72	SARC-F	4	②	12.3	46	7	16	434
Qian, 2022 (60)	Zheng zhou	Ccommunity	320	72.87	65	Finger-ring Test			②	20.63	45	45	21	209
						CC			②		48	51	18	203
Hu, 2021 (61)	Si chuan, Yun nan, Gui zhou, Xin jiang	Ccommunity	4509	63.5	64.18	MUAC	M 28.6cm	②	24.97	420	327	58	810
							F 27.5cm	②		497	498	151	1748
Mengli, 2021 (62)	Su zhou	Ccommunity	831	72.67	55.96	MUAC	M 26cm	②	13.6	30	48	5	283	F 26cm	②	49	89	29	298
						CC	M 33cm	②		28	34	7	297
							F 33cm	②		66	120	12	267
						Mengli, 2021 (63)	Su zhou	Ccommunity		1537	73.79	55.11	CC	M 33.7cm	②	12.27	38	80	5	360
F 33cm	②	76	153	13	351											
MUAC	M 25.9cm	②	37	71	6				369							
	F 26.5cm	②	63	160	26				344							
Min, 2018 (64)	He fei	Ccommunity	122	71.8	57.38	Ishii	M 105 F 120	① ①	30.33	14 17	10 14	2 4	26 35
Ping, 2019 (65)	Cheng du	Ccommunity	477	70.6	44.86	SARC-F	4	①	17	49	4	32	392
Mo, 2020 (66)	Chang sha	Ccommunity	1050	70.3	66.95	SARC-F	4	②	25.05	47	50	216	737
						SARC-CalF	11	②		125	63	138	724
						CC	M 34cm	②		61	59	23	204
							F 33cm	②		153	122	26	402
Chen, 2021 (67)	Si chuan	Ccommunity	941	NR	50.9	Ishii	M 95	②	18.38	65	69	27	301
							F 102	②		61	80	20	318
							M 105	②		60	54	32	316
							F 120	②		38	27	43	371
Jiaoling, 2023 (68)	Chang sha	Nursing home	386	80.3	56.74	Ishii	M 137	②	49.7	79	25	12	51
							F 161	②		79	32	22	86
							M 105	②		91	58	0	18
							F 120	②		100	90	1	28
Yang, 2018 (69)	Cheng du	Ccommunity	384	71.5	58.33	c-MSRA-7	30	①	15.89	53	195	8	128
								③	11.72	24	108	21	231
								⑤	25	52	80	44	208
								⑥	15.36	24	108	35	217
						c-MSRA-5	45	①	15.89	55	95	6	228
								③	11.72	32	118	13	221
								⑤	25	68	82	28	206
								⑥	15.36	53	97	6	228

①, AWGS 2014; ②, AWGS 2019; ③, EWGSOP 1; ④, EWGSOP 2; ⑤, IWGS; ⑥, FNIH; TP, true positives; FP, false positives; TN, true negative; FN, false negatives; MUAC, middle upper arm circumference; CC, calf circumference; T, total; M, male; F, female; NR, not report; c, Chinese version.

#### 3.1.3 Quality assessment

**Figure 2:**
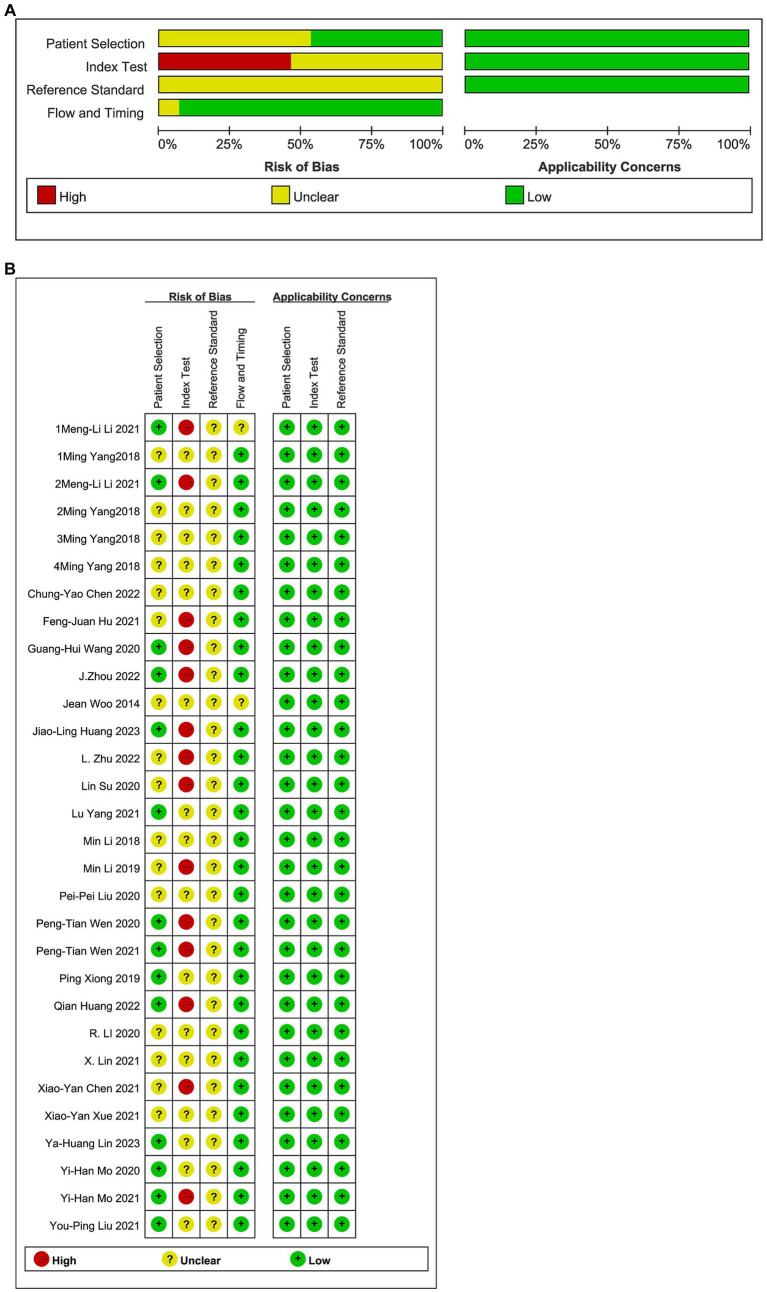
Results of the risk bias **(A)** The risk composition ratio; **(B)** The risk of each study.

The results of the risk–bias analysis of the included studies are shown in Figure 2. Of the included studies, 17 studies did not indicated whether the sample of patients included was continuous or not (41–52), only the timeline included in the patient sample was explained and were evaluated as unclear, five studies were not described and were evaluated as no (23, 53–57). Thirty studies did not describe whether the experiment was blinded or not and were evaluated as unclear (23, 41–43). Fourteen studies choose the test threshold and were evaluated as no (41, 43, 45, 50, 51, 53, 55, 58–60, 64, 66–68). It was difficult to determine whether the reference standard results were interpreted without knowledge of the results of the index test in 30 studies (41, 43, 45, 50, 51, 53, 55, 58–60, 64, 66–68). The number of patients enrolled was differs from the number of patients included in the 2 × 2 table of results in 1 study (45). Although patient selection, reference standard, flow and timing had a low to unclear risk of bias. The applicability concerns were low.

### 3.2 Pooled results for screening tool accuracy

**Figure 3:**
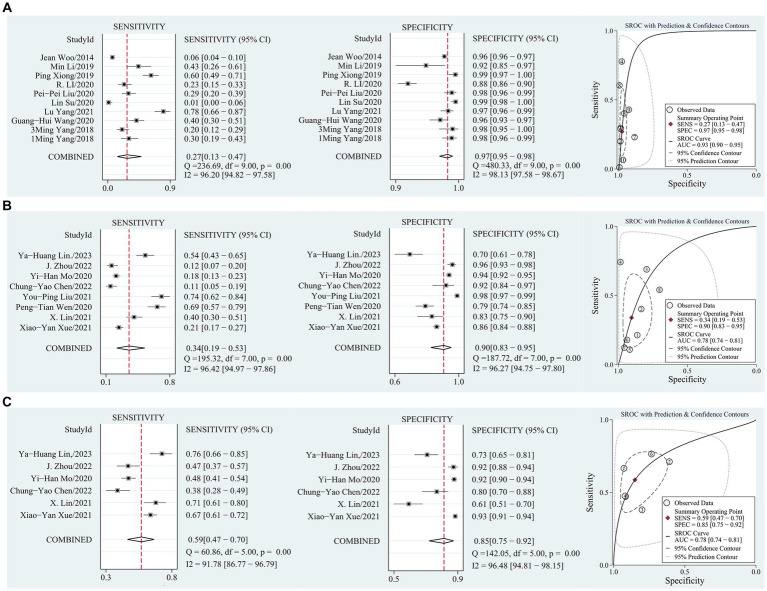
**(A)** Sensitivity and specificity coupled forest plot, SROC curve of SARC-F based on AWGS 2014; **(B)** Sensitivity and specificity coupled forest plot, SROC curve of SARC-F based on AWGS 2019; **(C)** Sensitivity and specificity coupled forest plot, SROC curve of SARC-CalF based on AWGS 2019.

Table 3 shows the pooled sensitivity, specificity, PLR, NLR, DOR, and AUC of the seven screening tools according to diagnostic criteria. Figure 3 shows the sensitivity and specificity of the coupled forest plots and SROC curves for SARC-F based on AWGS 2014 and AWGS 2019, and SARC-CalF based on AWGS 2019, respectively. The study for the MUAC was insufficient and not pooled for the accuracy. Meta-regression was used to account for sources of heterogeneity, and a subgroup analysis of the sources of heterogeneity was performed when the number of references met the criteria for the meta-analysis.

**Table 3:** Pooled results of the meta-analysis grouped by diagnostic criteria.

Diagnostic criteria	Screening tools	Sensitivity (95% CI)	I^2^	Specificity (95% CI)	I^2^	PLR (95% CI)	NLR (95% CI)	DOR (95% CI)	AUC
AWGS 2014①	SARC-F^†^	0.27(0.13–0.47)	96.20%	0.97(0.95–0.98)	98.13%	9.66(4.49–20.76)	0.75(0.59–0.95)	12.85(4.93–33.53)	0.93(0.90–0.95)
	SARC-CalF^‡^	0.53(0.47–0.59)	73.40%	0.88(0.86–0.90)	90.80%	5.06(2.43–10.57)	0.52(0.38–0.72)	9.81(3.53–27.27)	0.634
	MSRA-7^‡^	0.60(0.52–0.68)	16.10%	0.76(0.72–0.79)	88.40%	2.71(1.86–3.96)	0.51(0.42–0.62)	5.57(3.71–8.33)	——
	MSRA-5^‡^	0.68(0.60–0.75)	96.00%	0.75(0.71–0.79)	91.60%	3.12(2.64–3.70)	0.29(0.06–1.46)	11.10(3.10–39.74)	——
	Ishii^‡^	0.85(0.74–0.92)	0.00%	0.71(0.64–0.78)	0.00%	2.93(2.29–3.75)	0.22(0.12–0.38)	13.70(6.69–28.06)	——
AWGS 2019②	SARC-F^†^	0.34(0.19–0.53)	96.42%	0.90(0.83–0.95)	96.27%	3.53(1.65–7.51)	0.73(0.56–0.96)	4.82(1.81–12.88)	0.78(0.74–0.81)
	SARC-CalF^†^	0.59(0.57–0.70)	91.78%	0.85(0.75–0.92)	96.48%	3.91(2.38–6.40)	0.49(0.38–0.63)	8.03(4.38–14.70)	0.78(0.74–0.81)
	MSRA-5^‡^	0.61(0.54–0.69)	0.00%	0.74(0.69–0.79)	95.50%	2.09(0.85–5.12)	0.58(0.37–0.89)	3.62(0.96–13.67)	——
	CC^‡^	0.81(0.77–0.84)	26.50%	0.73(0.71–0.76)	89.40%	2.56(1.71–3.83)	0.29(0.20–0.42)	8.97(4.32–18.60)	0.8789
	Finger-ring Test^‡^	0.71(0.65–0.77)	0.00%	0.85(0.82–0.87)	0.00%	4.61(3.57–5.66)	0.34(0.28–0.42)	13.67(9.48–19.71)	0.9041
	Ishii^‡^	0.81(0.78–0.85)	98.50%	0.76(0.73–0.79)	99.40%	2.89(0.81–10.32)	0.15(0.04–0.61)	19.02(7.96–45.43)	0.876
EWGSOP 1③	SARC-F^‡^	0.10(0.08–0.13)	84.90%	0.96(0.96–0.97)	0.00%	3.39(1.71–6.72)	0.89(0.80–1.00)	3.82(1.72–8.47)	0.9782
	SARC-CalF^‡^	0.57(0.49–0.64)	28.00%	0.88(0.85–0.91)	77.50%	4.30(3.19–5.80)	0.51(0.43–0.60)	8.27(5.56–12.31)	——
	MSRA-7^‡^	0.53(0.45–0.62)	0.00%	0.72(0.68–0.76)	89.00%	2.11(1.31–3.40)	0.62(0.51–0.74)	3.43(1.84–6.41)	——
	MSRA-5^‡^	0.58(0.49–0.66)	80.20%	0.71(0.67–0.75)	94.90%	2.41(1.61–3.60)	0.55(0.42–0.71)	4.88(3.16–7.52)	——
EWGSOP 2④	SARC-F^‡^	0.25(0.18–0.33)	16.60%	0.97(0.95–0.98)	79.40%	8.18(2.35–28.41)	0.78(0.68–0.89)	10.53(2.68–41.32)	——
FNIH⑤	SARC-F^‡^	0.25(0.19–0.33)	27.60%	0.98(0.97–0.99)	0.00%	15.52(7.64–31.51)	0.76(0.68–0.85)	20.88(9.66–45.13)	——
	SARC-CalF^‡^	0.65(0.57–0.73)	75.90%	0.91(0.88–0.93)	86.20%	6.65(4.12–10.72)	0.39(0.26–0.58)	17.55(10.98–28.06)	——
	MSRA-7^‡^	0.51(0.42–0.59)	74.90%	0.72(0.68–0.76)	92.70%	1.95(0.78–4.89)	0.68(0.40–1.16)	2.87(0.68–12.12)	——
	MSRA-5^‡^	0.70(0.62–0.77)	95.20%	0.75(0.71–0.79)	91.90%	3.09(2.62–3.66)	0.29(0.07–1.25)	11.17(3.57–34.88)	——
IWGS⑥	SARC-F^‡^	0.08(0.07–0.10)	92.80%	0.97(0.96–0.97)	24.00%	4.98(1.34–18.56)	0.88(0.77–1.01)	5.67(1.35–23.84)	0.9995
	SARC-CalF^‡^	0.50(0.42–0.57)	70.50%	0.92(0.89–0.94)	91.20%	6.10(2.71–13.71)	0.56(0.48–0.66)	11.00(5.72–21.15)	——
	MSRA-7^‡^	0.54(0.47–0.61)	0.00%	0.77(0.73–0.80)	88.90%	2.55(1.44–4.53)	0.58(0.49–0.68)	4.38(2.12–9.05)	——
	MSRA-5^‡^	0.59(0.52–0.66)	90.00%	0.76(0.72–0.80)	88.90%	2.60(2.15–3.16)	0.51(0.34–0.77)	5.53(3.80–8.05)	——

①, Asian Working Group for Sarcopenia 2014; ②, Asian Working Group for Sarcopenia 2019; ③, European Working Group on Sarcopenia in Older People 2010; ④, European Working Group on Sarcopenia in Older People 2019; ⑤, International Working Group on Sarcopenia; ⑥, Foundation for the National Institutes of Health.

†, Use Stata 17.0 to merge accuracy; ‡, Use Meta-DiSc 1.4 to merge accuracy.

#### 3.2.1 Comparative results of the accuracy of the same screening tool based on different diagnostic criteria

**Figure 4:**
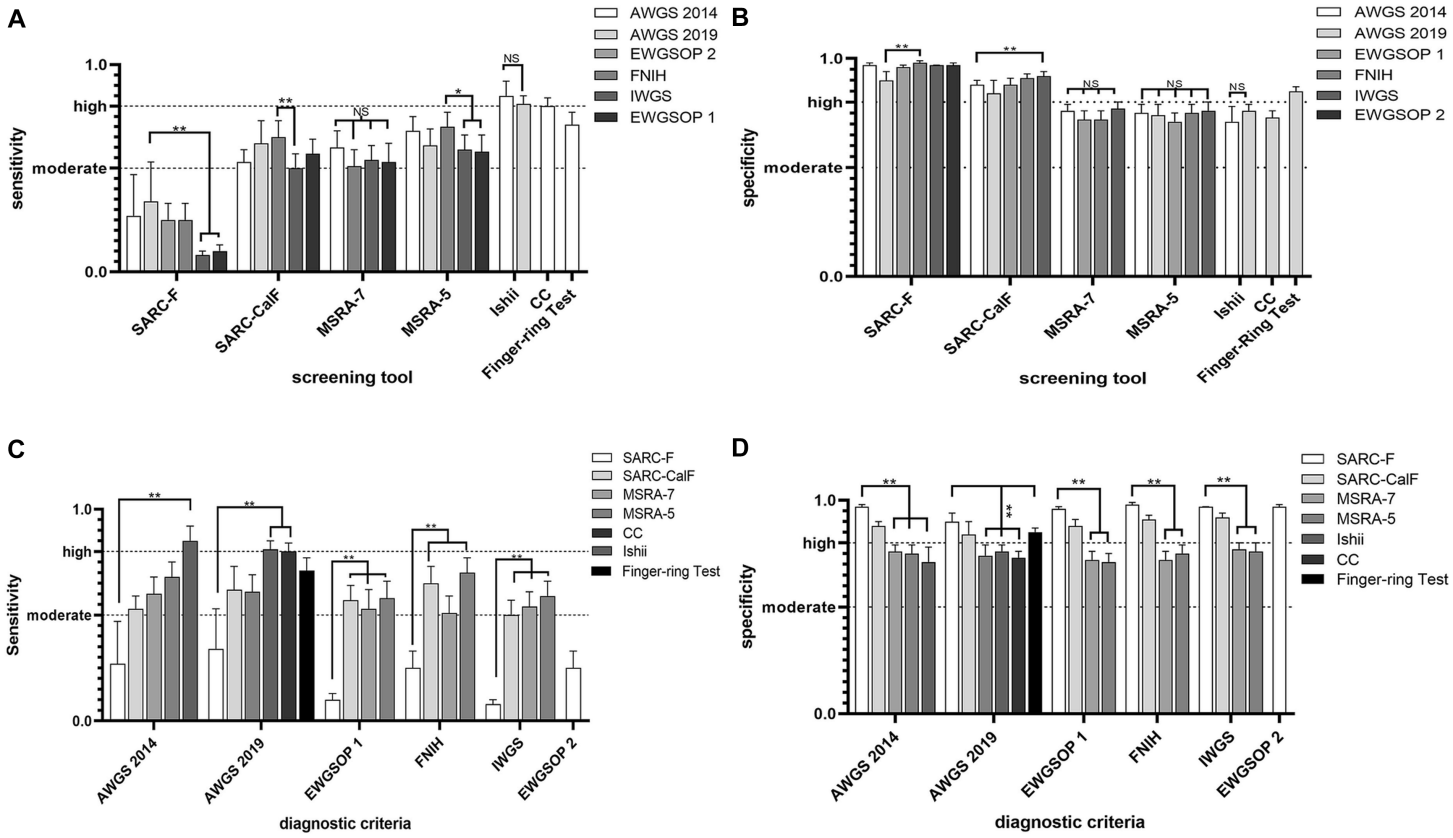
**(A)** Comparative results of sensitivity (highest and lowest), based on different diagnostic criteria; **(B)** Comparative results of specificity (highest and lowest), based on different diagnostic criteria. **(C)** Comparative results of sensitivity (highest and lowest), based on the same criteria. **(D)** Comparative results of specificity (highest and lowest), based on the same criteria.

Figures 4A,B showed the comparative results of the accuracy of the same screening tool based on different diagnostic criteria in 29 studies. Based on different diagnostic criteria, the sensitivity and specificity of SARC-F, SARC-CalF, and MSRA-5 were statistically different but remained at the same level. Overall, the SARC-F showed low sensitivity and high specificity, SARC-CalF showed moderate sensitivity and high specificity, MSRA-7 and MSRA-5 showed moderate sensitivity and specificity, Ishii showed high sensitivity and moderate specificity, CC showed high sensitivity and moderate specificity, and the Finger-ring Test showed moderate sensitivity and high specificity.

#### 3.2.2 Comparative results of the accuracy of different screening tools based on the same diagnostic criteria

Figures 4C,D show the comparative accuracy results of the different screening tools based on the same diagnostic criteria in 29 studies. In Chinese older adults, when AWGS 2014 as a diagnostic criterion for sarcopenia, Ishii had relatively high sensitivity (85%) but relatively low specificity (71%), SARC-F had relatively high specificity (97%) but relatively low sensitivity (27%). When AWGS 2019 as a diagnostic criterion for sarcopenia, CC and Ishii had relatively high sensitivity (81, 81%) but relatively low specificity (73, 76%); SARC-F had relatively high specificity (90%) but relatively low sensitivity (34%). When EWGSOP 1 as a diagnostic criterion for sarcopenia, MSRA-5 had relatively high sensitivity (58%), but relatively low specificity (71%), and SARC-F had relatively high specificity (96%), but relatively low sensitivity (10%). When FNIH as a diagnostic criterion for sarcopenia, MSRA-5 had relatively high sensitivity (70%), but relatively low specificity (75%), and SARC-F had relatively high specificity (98%), but relatively low sensitivity (25%); When IWGS was used as a diagnostic criterion for sarcopenia, MSRA-5 had relatively high sensitivity (59%), but relatively low specificity (76%), and SARC-F had relatively high specificity (97%), but relatively low sensitivity (8%).

#### 3.2.3 Exploring heterogeneity in the accuracy of different screening tools combined results based on different diagnostic criteria

Using AWGS 2014 as the diagnostic criterion, I^2^ showed high heterogeneity for pooled sensitivity of SARC-F and MSRA-5 (I^2^ = 96.2 and 96.0%, respectively) and specificity (I^2^ = 88.4–98.13%) for all screening tools with the exception of Ishii. Using AWGS 2019 as the diagnostic criterion, I^2^ showed high heterogeneity for pooled sensitivity of SARC-F, SARC-CalF and Ishii (I^2^ = 91.78–98.5%) and specificity (I^2^ = 89.4–99.4%) for all screening tools with the exception of Finger-ring Test. Using EWGSOP 1 as the diagnostic criterion, I^2^ showed high heterogeneity for pooled sensitivity of SARC-F and MSRA-5 (I^2^ = 84.9 and 80.2%, respectively) and specificity of MSRA-7 and MSRA-5 (I^2^ = 89.0 and 94.9%, respectively). Using FNIH as the diagnostic criterion, I^2^ showed high heterogeneity for pooled sensitivity of MSRA-5 (I^2^ = 95.2%) and specificity of SARC-CalF, MSRA-7 and MSRA-5 (I^2^ = 86.2–92.7%). Using IWGS as the diagnostic criterion, I^2^ showed high heterogeneity for pooled sensitivity of SARC-F and MSRA-5 (I^2^ = 92.8 and 90.0%, respectively) and specificity of SARC-CalF, MSRA-7 and MSRA-5 (I^2^ = 88.9–91.2%).

Owing to the insufficient number of included references, we only performed a meta-regression of the screening tools using AWGS 2014 and AWGS 2019 as diagnostic criteria to explain the sources of heterogeneity. When the number of references met the criteria for meta-analysis, subgroup analysis was performed on the sources of heterogeneity.

Among the various potential covariates, SARC-F used the diagnostic criteria of AWGS 2014 (n = 10), meta-regression showed a statistically significant difference in specificity for the region (eastern vs. western), with a specificity of 0.98 (95% CI, 0.96–0.99) vs. 0.96 (95% CI, 0.93–0.99), (*p* = 0.03). CC used the diagnostic criteria of AWGS 2019 (*n* = 6), meta-regression showed that sex (female vs. male) was a significant factor associated with study heterogeneity, with a statistically significant difference in sensitivity of 0.75 (95% CI, 0.69–0.81) vs. 0.85 (95% CI, 0.81–0.88), (*p* = 0.00).

Because the cutoff values for CC and Ishii screening for sarcopenia were sex-differentiated, they were analyzed in the sex subgroups (Table 4).

**Table 4:** The pooled results of the CC and Ishii meta-analysis grouped by definition and sex.

Diagnostic criteria	Screening tools	Sex	Sensitivity (95% CI)	I^2^	Specificity (95% CI)	I^2^	PLR (95% CI)	NLR (95% CI)	DOR (95% CI)	AUC
②	CC^†^	Male	0.75(0.68–0.80)	0.00%	0.74(0.64–0.81)	67.36%	2.58(2.01–4.06)	0.34(0.26–0.46)	8.34(4.57–15.20)	0.79(0.75–0.82)
		Female	0.85(0.81–0.88)	0.00%	0.67(0.61–0.73)	83.04%	2.60(2.11–3.20)	0.23(0.17–0.30)	11.45(7.39–17.74)	0.86(0.83–0.89)
①	Ishii^‡^	Male	0.88(0.71–0.96)	0.00%	0.71(0.60–0.80)	0.00%	2.99(2.11–4.25)	0.18(0.07–0.44)	17.13(5.44–53.91)	——
		Female	0.80(0.64–0.91)	0.00%	0.70(0.60–0.79)	0.00%	2.64(1.88–3.69)	0.29(0.15–0.54)	9.20(3.79–22.32)	——
②	Ishii^‡^	Male	0.85(0.80–0.90)	96.50%	0.73(0.69–0.77)	98.30%	2.32(0.92–5.84)	0.14(0.03–0.66)	14.41(7.14–29.12)	0.8579
		Female	0.77(0.71–0.82)	97.50%	0.78(0.75–0.82)	99.10%	3.68(0.68–19.98)	0.23(0.07–0.78)	16.36(8.89–30.09)	0.8852

①, Asian Working Group for Sarcopenia 2014; ②, Asian Working Group for Sarcopenia 2019; PLR, positive likelihood ratio; NLR, negative likelihood ratio; DOR, diagnostic odds ratio; AUC, area under the curve.

†, Use Stata 17.0 to merge accuracy; ‡, Use Meta-DiSc 1.4 to merge accuracy.

### 3.3 Publication bias

**Figure 5:**
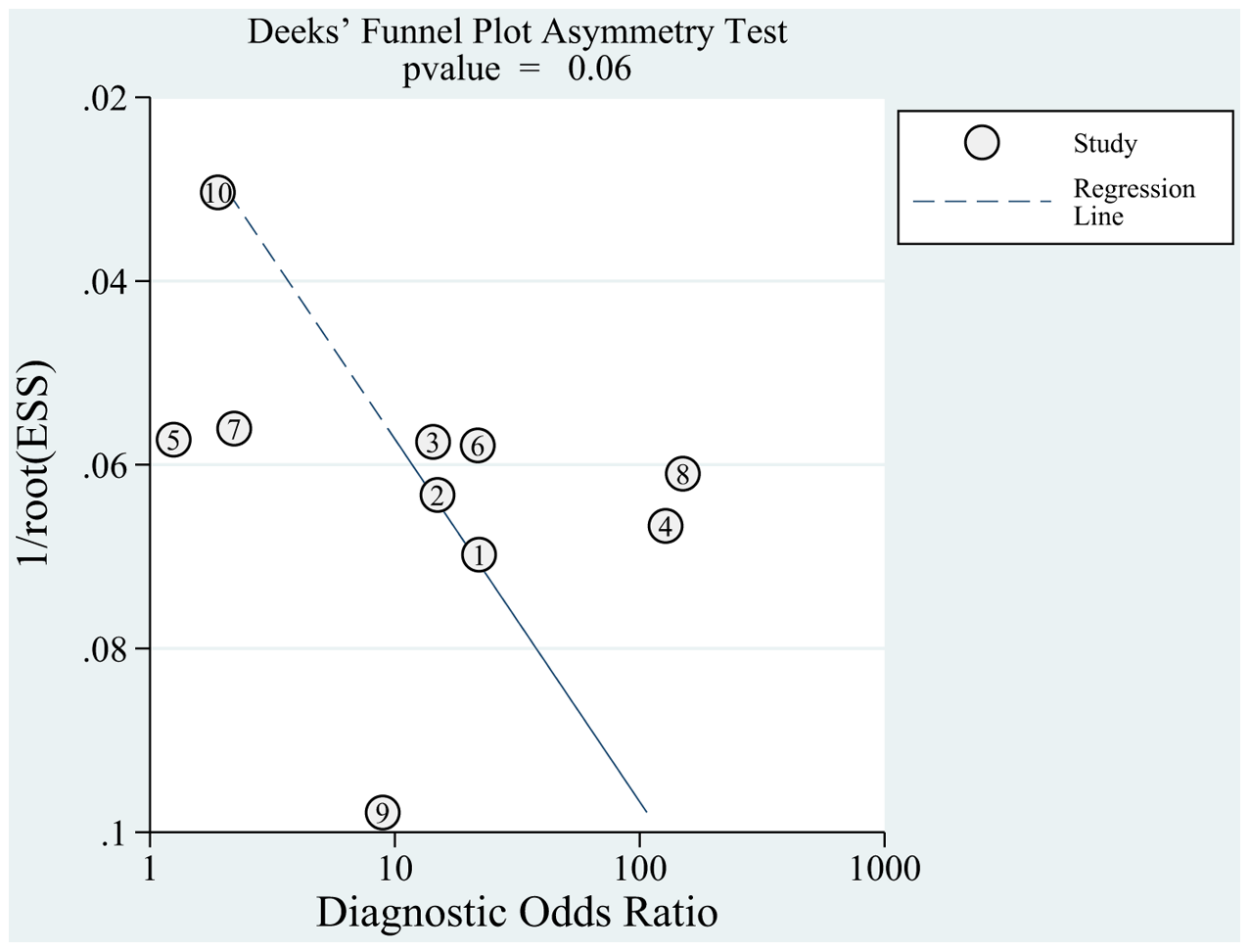
Funnel plot with superimposed regression line.

Given the number of included references, Deeks’ funnel plot asymmetry test was applied separately to references based on AWGS 2014 (*n* = 10) (23, 41–45, 49–51, 65) to estimate publication bias. Deeks’ funnel plot (Figure 5) did not reveal any evidence of publication bias (*p* = 0.06).

## 4 Discussion

### 4.1 Prevalence of sarcopenia in China

Using different diagnostic criteria, the prevalence of sarcopenia in older Chinese adults in this study ranged from 10.63 to 22.94%, similar to the results of a previous study on the prevalence of sarcopenia in older Chinese adults (70, 71). The prevalence of using the AWGS 2019 diagnostic criteria (22.94%) was much higher than that of the AWGS 2014 diagnostic criteria (11.22%) because the AWGS 2019 diagnostic criteria increased the cutoff points for gait speed and male grip strength. In a global meta-analysis of the prevalence of sarcopenia that included 151 studies (72), the prevalence of diagnostic criteria using EWGSOP 1 was 22%, which was significantly different from the prevalence using EWGSOP 1 in this study (10.63%) and partially influenced by the small number of relevant references included in this study. Furthermore, differences in the prevalence of sarcopenia may be influenced by the population (the study and reference populations), and different methods of assessment and race may play a role when the reference and study populations are mismatched (73, 74).

### 4.2 Heterogeneity

The screening tools for sarcopenia included in this study, except for the CC, Ishii, and Finger-ring Test, generally showed low to moderate sensitivity and moderate to high specificity, and the screening tools have poor sensitivity for screening sarcopenia in older Chinese adults. The pooled results for accuracy of some screening tools showed high heterogeneity, and exploration of heterogeneity using meta-regression showed that regional and sex differences affected the accuracy of the screening tools. A meta-analysis based on global validation of the accuracy of SARC-F screening for sarcopenia showed that the accuracy of SARC-F for screening for sarcopenia in Asian and non-Asian countries differed (75). Expert consensus on the diagnosis and treatment of sarcopenia in older Chinese adults suggests that western China is at a higher altitude than eastern China and that lifestyle and environment are the main factors affecting the prevalence of sarcopenia (76). In addition, the prevalence was slightly higher in males than in females. This shows that the same screening tool cannot be applied simultaneously in the eastern and western regions of China and that the screening tool should establish corresponding cutoff values for males and females.

### 4.3 The accuracy of screening tools

In older Chinese adults, the accuracy of the same screening tool under different diagnostic criteria varies but remains at the same level. Based on different diagnostic criteria, SARC-F shows high specificity, but its low sensitivity is a major weakness as a screening tool for sarcopenia; that is, SARC-F has a low rate of misdiagnosis when screening for sarcopenia, but a high rate of misdiagnosis (32, 33). Barbosa-Silva et al. believed that low sensitivity was due to the omission of low muscle mass in the questionnaire, in which CC was added to the SARC-F questionnaire to increase sensitivity (12). The sensitivity of the SARC-CalF test was higher than that of the SARC-F test. However, an increase in sensitivity led to a decrease in specificity. This is similar to the results of a Korean study based on 2,123 community-dwelling older adults (mean age, 75.9 ± 3.9 years) (77). However, the sensitivity of the SARC-CalF test is less than perfect. MSRA-5, which is based on MSRA-7 with removed food intake questions, increases the proportion of weight and physical activity level scores; compared to MSRA-7, MSRA-5 improves sensitivity while maintaining the same specificity, indicating that weight loss and low physical activity levels are predictors of sarcopenia in older Chinese adults. This conjecture was confirmed by Van Kan et al. (78, 79). Compared to other screening tools, CC, Ishii, and Finger-ring Test performed better in screening for sarcopenia in older Chinese adults. However, they are not perfect screening tools for sarcopenia because missing sarcopenia may make these high-risk individuals prone to adverse health outcomes (80, 81), A high sensitivity of SARC-CalF, high sensitivity and moderate specificity of CC and Ishii, and moderate sensitivity and high specificity of the Finger-ring Test indicate that CC may be a simple but valuable screening tool for sarcopenia or a valid indicator of a high correlation with muscle mass, and may improve screening accuracy when combined with other relevant parameters as a screening tool. The findings from the present study are consistent with those from earlier studies (82–84).

### 4.4 Diagnostic criteria of sarcopenia for the older adults in China

AWGS 2019 considers ethnic differences in different populations and is more applicable to the diagnosis of sarcopenia in Asians than other diagnostic criteria. AWGS 2019 also introduces the concept of “probable sarcopenia” to facilitate timely interventions (20). Aging is an important risk factor associated with decreased muscle function (85, 86), and with timely intervention it is possible to improve physical function and slow the decreases in muscle quantity and quality (8, 9, 87). Therefore, it is not too late for older adults to undergo screening for sarcopenia or interventions. Although the AWGS 2019 consensus recommends the use of SARC-F and SARC-CalF for sarcopenia screening, the low sensitivity of the screening tool leads to a higher risk of missed diagnoses. We believe that CC and Ishii have better sarcopenia screening performance and that SARC-F and SARC-CalF should be used with caution in screening for sarcopenia.

### 4.5 Areas for further research

As the accuracy of screening tools is affected by regional differences, it is necessary to improve or develop screening tools for sarcopenia in different regions of China. The pooled results of the accuracy of the screening tool for sarcopenia showed that there is room for improvement in the sensitivity of the screening tool. CC was strongly correlated with muscle mass and its inclusion should be considered in the future to improve the accuracy of the screening tools. Further experimental studies are required to validate this screening tool for sarcopenia in Chinese older adults.

## 5 Strengths and limitations

This study compared the accuracy of sarcopenia screening tools based on different diagnostic criteria in older Chinese adults. We included several studies with an “unclear” to “high” risk of bias in the experiments to be evaluated, and the selection of the cutoff value to optimize sensitivity and specificity may lead to increased screening accuracy which may have an impact on the accuracy of the study results. The cutoff values of MUAC have not been standardized, and the number of references is insufficient for meta-analysis; this study only reported the accuracy range of MUAC screening for sarcopenia in Chinese older adults using different cutoff values. Due to the small number of relevant references, meta-regression could not be performed to explain the existence of partial heterogeneity, which may have affected the objectivity of the pooled results. However, further research is required to confirm the accuracy of these screening tools.

## 6 Conclusion

Comparisons of the accuracy of the same screening tools with different diagnostic criteria showed that the AWGS 2019 diagnostic criteria were more appropriate for the diagnosis of sarcopenia in older Chinese adults. Although there are screening tools that showed higher DOR and AUC using the diagnostic criteria of AWGS 2019, CC and Ishii have relatively high sensitivity. Considering the importance of high sensitivity in sarcopenia screening, CC and Ishii score are recommended for sarcopenia screening in older Chinese adults.

## Data availability statement

The original contributions presented in the study are included in the article/Supplementary material, further inquiries can be directed to the corresponding authors.

## Author contributions

SQ: Writing – review & editing, Conceptualization, Formal analysis, Methodology, Validation, Writing – original draft. SZ: Formal analysis, Methodology, Validation, Writing – original draft, Writing – review & editing. ML: Formal analysis, Validation, Writing – original draft, Writing – review & editing. SC: Data curation, Visualization, Writing – review & editing. LL: Data curation, Visualization, Writing – review & editing. SL: Data curation, Visualization, Writing – review & editing. FJ: Conceptualization, Methodology, Supervision, Writing – review & editing. JZ: Conceptualization, Methodology, Supervision, Writing – review & editing.

## Figures and Tables

**Table:** 

AWGS	Asian Working Group on Sarcopenia
EWGSOP	European Working Group on Sarcopenia in Older People
IWGS	International Working Group on Sarcopenia
FNIH	Foundation for the National Institutes of Health
SARC-CalF	SARC-F combined calf circumference
MSRA	Mini Sarcopenia Risk Assessment
CC	Calf Circumference
MUAC	middle upper arm circumference
PLR	positive likelihood ratio
NLR	negative likelihood ratio
DOR	diagnostic odds ratio
AUC	area under the curve
TP	true positives
FP	false positives
TN	true negative
FN	false negatives
M	male
F	female
T	total

## Footnotes

^1^ https://www.bris.ac.uk/quadas
