# Supplementary material for: The accuracy of screening tools for sarcopenia in older Chinese adults: a systematic review and meta-analysis
Source: Front Public Health. 2024 Feb 5;12:1310383. doi: 10.3389/fpubh.2024.1310383 (PMC10876058; doi:10.3389/fpubh.2024.1310383)
Supplement: Supplementary file 1 [file Data_Sheet_1.docx]

Supplementary Material

| **Databases** | **Search terms** | **Results** |
| --- | --- | --- |
| Web of Science | TS=(("sarcopenia screening tool" OR "screening tool" OR "SARC-F" OR "SARC-CalF" OR "MSRA" OR "Mini Sarcopenia Risk Assessment" OR "MSRA-7" OR "MSRA-5" OR "MUCA" OR "Mid Upper arm circumference" OR "CC" OR "Calf circumference" OR "Ishii" OR "Yubi-wakka" OR "finger ring test"))) AND TS=("sarcopenia" OR "muscle loss" | 1246 |
| **PubMed** | "sarcopenia screening tool" OR "screening tool" OR "SARC-F" OR "SARC-CalF" OR "MSRA" OR "Mini Sarcopenia Risk Assessment" OR "MSRA-7" OR "MSRA-5" OR "MUCA" OR "Mid Upper arm circumference" OR "CC" OR "Calf circumference" OR "Ishii" OR "Yubi-wakka" OR "finger ring test") AND ("sarcopenia" OR "muscle loss"[MeSH Terms] | 928 |
| **Embase** | 1、'sarc-f':ab,ti OR 'sarc-calf':ab,ti OR 'msra':ab,ti OR 'msra-7':ab,ti OR 'msra-5':ab,ti OR 'muca':ab,ti OR 'mid upper arm circumference':ab,ti OR 'cc':ab,ti OR 'calf circumference':ab,ti OR 'ishii':ab,ti OR 'yubi-wakka':ab,ti OR 'finger ring test':ab,ti OR 'screening tool':ab,ti2、 (2010:py OR 2011:py OR 2012:py OR 2013:py OR 2014:py OR 2015:py OR 2016:py OR 2017:py OR 2018:py OR 2019:py OR 2020:py OR 2021:py OR 2022:py OR 2023:py) #1 AND #2 AND #3 | 1140 |
| **China Knowledge Resource Integrated Database (CNKI)** | FT=(肌少症 OR 少肌症 OR 肌肉减少症 OR 骨骼肌减少症 OR 肌肉流失) and FT=(筛查工具 OR SARC-F OR SARC-CalF OR MUCA OR 中上臂围 OR 小腿围 OR 指环测试 OR Yubi-wakka OR MSRA OR 迷你肌少症风险评估 OR Ishii) | 345 |
| **WANFANG DATA** | (主题:(肌少症 OR 骨骼肌减少症 OR 少肌症 OR 肌肉减少症 OR 肌肉流失) and 全部:(筛查工具 OR SARC-F OR SARC-CalF OR MUCA OR 中上臂围 OR CC OR 小腿围 OR 指环测试 OR Yubi-wakka OR MSRA OR 迷你肌少症风险评估 OR Ishii)) and Date:2010-2023 | 802 |

# **Literature search strategies**

# **Content of screening tools**

## SARC-F and SARC-CalF

| **Components** | **Questions** | **SARC-F scoring** | **SARC-CalF scoring** |
| --- | --- | --- | --- |
| Strength | How much difficulty do you have in lifting and carrying 10 pounds? | None = 0  Some = 1  A lot or unable = 2 | None = 0  Some = 1  A lot or unable = 2 |
| Assistance in walking | How much difficulty do you have walking across a room? | None = 0  Some = 1  A lot, use aids, or unable = 2 | None = 0  Some = 1  A lot, use aids, or unable = 2 |
| Rise from a chair | How much difficulty do you have transferring from a chair or bed? | None = 0  Some = 1  A lot or unable without  help = 2 | None = 0  Some = 1  A lot or unable without  help = 2 |
| Climb stairs | How much difficulty do you have climbing a flight of 10 stairs? | None = 0  Some = 1  A lot or unable = 2 | None = 0  Some = 1  A lot or unable = 2 |
| Falls | How many times have you fallen in the past year? | None = 0  1 - 3 falls = 1  4 or more falls = 2 | None = 0  1 - 3 falls = 1  4 or more falls = 2 |
| Calf circumference |  |  | Females:  >33 cm = 0  ≤33 cm = 10  Males:  >34 cm = 0  ≤34 cm = 10 |

## MSRA-5 and MSRA-7

| **Questions** | **MSRA-7**  **score** | **MSRA-5**  **score** |
| --- | --- | --- |
| How old are you?   - ≥70 y - <70 y | 0  5 | 0  5 |
| Were you hospitalized in the last year?   - Yes, and more than 1 hospitalization - Yes, 1 hospitalization - No | 0  5  10 | 0  10  15 |
| What is your activity level?   - I’m able to walk <1000 m - I’m able to walk more than 1000 m | 0  5 | 0  15 |
| Do you eat 3 meals per day regularly?   - No, up to twice per week I skip a meal (e.g., I skip   breakfast or I have only milky coffee or soup for dinner)   - Yes | 0  5 | 0  15 |
| Do you consume any of the following?   - Milk or dairy products (e.g., yogurt, cheese), but not every day - Milk or dairy products (e.g., yogurt, cheese), at least once per day | 0  5 | —  — |
| Do you consume any of the following?   - Poultry, meat, fish, eggs, legumes, ragout, or ham, but not every day - Poultry, meat, fish, eggs, legumes, ragout, or ham, at least once per day | 0  5 | —  — |
| Did you lose weight in the last year?   - >2 kg - 2 kg | 0  5 | 0  10 |

## Ishii

**Male:** 0.62 × (age -  64) - 3.09 × (grip strength - 50) - 4.64 × (calf circumference - 42);

Diagnosed with sarcopenia：Ishii score ≥ 105.

**Female:** 0.80 × (age - 64) - 5.09 × (grip strength - 34) - 3.28 × (calf circumference - 42).

Diagnosed with sarcopenia：Ishii score ≥ 120.

## Calf circumference (CC)

## **Male:** <34 cm;

**Female:** <33 cm.

## Middle upper arm circumference

There is no uniform critical value yet.

## Finger-ring Test/ Yubi-Wakka

The test was carried out as shown in Supplementary Figure 1, all procedures were carried out with the participant in a seated position without the use of any instrument.


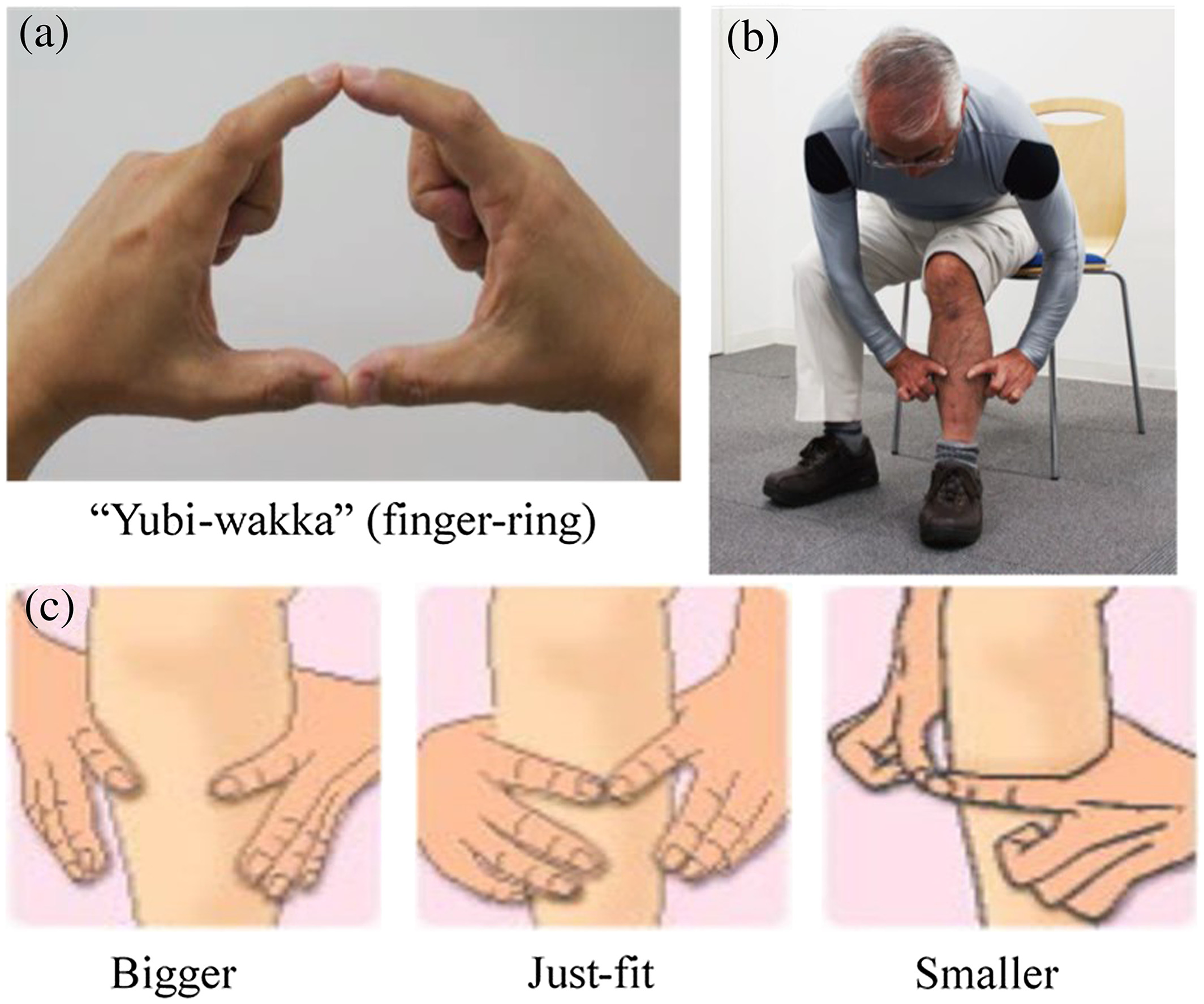


Supplementary Figure 1. (a)making a ring with the index fingers and thumbs of both hands; (b) gently circling the thickest part of the non-dominant calf of the participant's leg, which is bent at a 90°angle, and; (c) checking whether or not the non-dominant calf circumference is “bigger,” “just fits” or “smaller” as compared with the finger-ring circumference.
